# Association between maternal preeclampsia and the risk of neonatal sepsis: a systematic review and meta-analysis

**DOI:** 10.3389/fped.2026.1813564

**Published:** 2026-04-13

**Authors:** Chang Zhou, Dongyang Liu, Junxia Wang

**Affiliations:** Department of Pediatrics, General Hospital of Western Theater Command, Chengdu, China

**Keywords:** gestational age, meta-analysis, neonatal sepsis, preeclampsia, risk factor

## Abstract

**Background:**

Background: Preeclampsia is a common hypertensive disorder of pregnancy associated with adverse maternal and neonatal outcomes, while neonatal sepsis remains a leading cause of neonatal morbidity and mortality worldwide. However, the association between maternal preeclampsia and neonatal sepsis remains uncertain, with inconsistent findings across studies. We conducted a systematic review and meta-analysis to evaluate this relationship.

**Methods:**

PubMed, Embase, and Web of Science were searched from inception to January 05, 2026, including full-text articles published in English. Observational studies including neonates (≤28 days) born to women with and without preeclampsia, with gestational age (GA) at delivery matched or adjusted, were eligible. Risk ratios (RRs) were pooled using random-effects models accounting for between-study heterogeneity. The certainty of evidence for the outcome was assessed using the GRADE framework.

**Results:**

Eleven cohort studies comprising 1,513,008 neonates (65,848 exposed to preeclampsia) and 24,925 cases of neonatal sepsis were included. Overall, preeclampsia was associated with a modestly increased risk of neonatal sepsis (RR: 1.27, 95% CI: 1.02–1.56; *p* = 0.03), although substantial heterogeneity was present (*I*^2^ = 88%). Sensitivity analyses showed broadly consistent estimates (RR range: 1.18–1.32). Excluding the largest study reduced heterogeneity (*I*^2^ = 34%) while the association remained statistically significant (RR: 1.18, 95% CI: 1.05–1.32). Subgroup analyses did not demonstrate statistically significant differences across study design, sample size, GA at delivery, exposure or outcome definitions (all *p* for subgroup difference >0.05). Stronger associations were observed in studies with lower NOS scores (7–8 vs. 9; *p* = 0.04). Meta-regression suggested that sample size and study quality may partially explain between-study heterogeneity. The overall certainty of evidence for this association was rated as low according to the GRADE framework.

**Conclusions:**

Maternal preeclampsia may be associated with a modest increase in the risk of neonatal sepsis. These findings suggest that neonates born to mothers with preeclampsia may warrant closer clinical surveillance for early signs of infection. However, substantial heterogeneity and the low certainty of evidence warrant cautious interpretation, and further well-designed studies are needed to clarify the clinical significance and underlying mechanisms of this association.

**Systematic Review Registration:**

identifier CRD420261320994.

## Introduction

Neonatal sepsis remains a major cause of morbidity and mortality worldwide, particularly in preterm and low-birth-weight infants (1, 2). Neonatal sepsis remains a major cause of neonatal morbidity and mortality worldwide, particularly in low- and middle-income countries, where it contributes substantially to early-life deaths and long-term adverse outcomes (3). Established risk factors include prematurity, low birth weight, prolonged rupture of membranes, chorioamnionitis, invasive procedures, central catheterization, mechanical ventilation, and maternal infections (4, 5). Among these, gestational age (GA) is one of the strongest determinants, as immaturity of the innate and adaptive immune systems renders preterm neonates particularly vulnerable to infection (6, 7). However, risk stratification based solely on neonatal characteristics may be insufficient, and increasing attention has been directed toward maternal conditions that may predispose offspring to infectious complications (8). Identifying novel maternal risk factors could improve early surveillance and targeted preventive strategies in high-risk neonates.

Preeclampsia is a hypertensive disorder of pregnancy, classically defined as new-onset hypertension (blood pressure ≥140/90 mmHg after 20 weeks of gestation) accompanied by proteinuria or signs of maternal organ dysfunction (9). It complicates approximately 2%–8% of pregnancies worldwide and remains a leading cause of maternal and perinatal morbidity and mortality (10, 11). Beyond its well-recognized association with preterm delivery, fetal growth restriction, and placental insufficiency (12), preeclampsia may influence neonatal immune competence through mechanisms such as chronic intrauterine inflammation, oxidative stress, endothelial dysfunction, and altered placental transfer of immune mediators (13, 14). Previous observational studies, including both cohort and case–control designs, have evaluated the association between maternal preeclampsia and neonatal sepsis. However, their findings have been inconsistent (15–25). A major methodological limitation across these studies is the variable handling of gestational age (GA), a key determinant of neonatal sepsis risk that is also closely linked to preeclampsia (7, 26). Inadequate adjustment for GA in some studies raises concerns about residual confounding, while differences in study populations, exposure definitions, and outcome ascertainment further contribute to heterogeneity and limit comparability (15–25). To address these limitations, we conducted a systematic review and meta-analysis focusing specifically on studies in which GA at delivery was matched or adjusted. By minimizing confounding from prematurity, this approach aims to provide a more reliable estimate of the independent association between maternal preeclampsia and neonatal sepsis. Clarifying this relationship may have important clinical implications, including informing risk stratification and guiding early surveillance and preventive strategies for neonates at increased risk of infection.

## Methods

The meta-analysis was carried out in accordance with established methodological guidance, following the principles outlined in the PRISMA 2020 statement (27) and the Cochrane Handbook for Systematic Reviews and Meta-Analyses (28), encompassing protocol planning, study selection, data extraction, statistical analysis, and reporting. The study protocol was registered prospectively in the PROSPERO database (registration number: CRD420261320994).

### Database search

We carried out a comprehensive literature search across PubMed, Embase, and Web of Science to identify eligible studies for inclusion. The search strategy was constructed using the combination of the following terms: (1) “preeclampsia” OR “pre-eclampsia” OR “preeclamptic”; (2) “neonatal” OR “newborn” OR “infant” OR “infantile” OR “neonate”; and (3) “sepsis” OR “septic” OR “septicemia”. Only full-text, peer-reviewed articles published in English and conducted in human populations were considered eligible. We also manually examined the reference lists of relevant reviews and original studies to capture additional potentially eligible reports. Each database was searched from inception through January 05, 2026. The complete search strategies for all databases are provided in Supplemental File 2.

### Study inclusion and exclusion criteria

The selection of studies was guided by the PICOS principle:

Population (P): Neonates (≤28 days of life) born to women with and without preeclampsia, at least matched or adjusted for GA at delivery.
Exposure (I): Maternal preeclampsia diagnosed according to established clinical criteria (e.g., ACOG, ISSHP, or study-defined diagnostic standards) or consistent with the criteria used in the original studies.
Comparator (C): Neonates born to mothers without preeclampsia (normotensive pregnancies).
Outcome (O): Neonatal sepsis, diagnosed according to the criteria used in the original studies, including culture-proved sepsis, clinically diagnosed sepsis, or sepsis as evidenced by the database codes.
Study Design (S): Observational studies with longitudinal follow-up, including prospective or retrospective studies, or nested case-control studies.

Studies were excluded if they were reviews, meta-analyses, editorials, letters, conference abstracts lacking sufficient data, case reports, or case series; if they did not provide extractable data on the association between maternal preeclampsia and neonatal sepsis; if hypertensive disorders of pregnancy were reported without separate data for preeclampsia; if neonatal sepsis was not clearly defined; if duplicate or overlapping populations were identified (in which case the study with the largest or most comprehensive sample was included); or if the study was conducted in animals or focused exclusively on maternal sepsis rather than neonatal outcomes.

### Study quality evaluation and data extraction

Two reviewers independently performed the literature search, screened eligible studies, assessed study quality, and extracted relevant data. Any disagreements were resolved through discussion, and when necessary, by consulting the corresponding author. Study quality was appraised using the Newcastle–Ottawa Scale (NOS) (29). The NOS examines methodological rigor across selection, comparability, and outcome ascertainment domains. Total scores vary from 1 to 9, and studies achieving ≥7 points were considered to be of high quality. Extracted data included study characteristics (first author, publication year, country, and study design), participant characteristics (numbers of included neonates, sex distribution, mean maternal age at birth, mean GA at delivery, and mean birth weight of neonates born to women with preeclampsia), exposure assessment (diagnostic criteria for preeclampsia and number of neonates born to women with preeclampsia), outcome validation (diagnostic methods for neonatal sepsis and number of cases with neonatal sepsis), and covariates matched or adjusted when the association between preeclampsia and the risk of neonatal sepsis was analyzed. No imputation of missing or incomplete outcome data was performed. Only studies with sufficient data to calculate effect estimates were included in the meta-analysis.

### Statistical analyses

The association between preeclampsia and the risk of neonatal sepsis was evaluated by combining risk ratios (RRs) and their corresponding 95% confidence intervals (CIs), comparing individuals born to women with and without preeclampsia. When necessary, effect estimates and standard errors were derived from reported 95% CIs or *p* values. All estimates were log-transformed before pooling to enhance normal distribution assumptions and stabilize variances (28). To evaluate variability across studies, we applied the Cochrane Q test and calculated the *I*^2^ statistic (30). *I*^2^ values below 25% were classified as low heterogeneity, 25%–75% as moderate, and above 75% as high heterogeneity. Pooled effect estimates were calculated using a random-effects model to accommodate variability across studies (28). We performed leave-one-out sensitivity analyses, sequentially excluding individual studies to assess the robustness of the findings (31). To identify possible sources of between-study variability, we performed predefined subgroup analyses stratified by sample size, study design (prospective or retrospective), mean GA at delivery, criteria for the diagnosis of preeclampsia, mean birth weight of neonates born to women with preeclampsia, methods for the diagnosis of neonatal sepsis, and NOS scores. For subgroup analyses, continuous study-level variables (e.g., sample size, mean gestational age, and mean birth weight) were dichotomized using the median values across the included studies as cutoffs. This approach was adopted to achieve a balanced number of studies within each subgroup and to facilitate meaningful comparisons. These subgroup analyses were exploratory in nature. Moreover, the univariate meta-regression analysis was also performed to evaluate if the study characteristics could significantly modify the association between preeclampsia and risk of neonatal sepsis (28), including sample size, mean GA at delivery, mean birth weight of neonates born to women with preeclampsia, and NOS scores. To assess potential publication bias, we inspected funnel plots for asymmetry and conducted Egger’s regression analysis (32). A two-tailed *p* value <0.05 was considered statistically significant. All statistical analyses were performed using RevMan (version 5.3; Cochrane Collaboration, Oxford, UK) and Stata (version 17.0; StataCorp, College Station, TX, USA). The certainty of evidence for the primary outcome was assessed using the GRADE (Grading of Recommendations, Assessment, Development and Evaluation) framework, considering risk of bias, inconsistency, indirectness, imprecision, and publication bias (28). The overall certainty of evidence was categorized as high, moderate, low, or very low.

## Results

### Database search results

**Figure 1:**
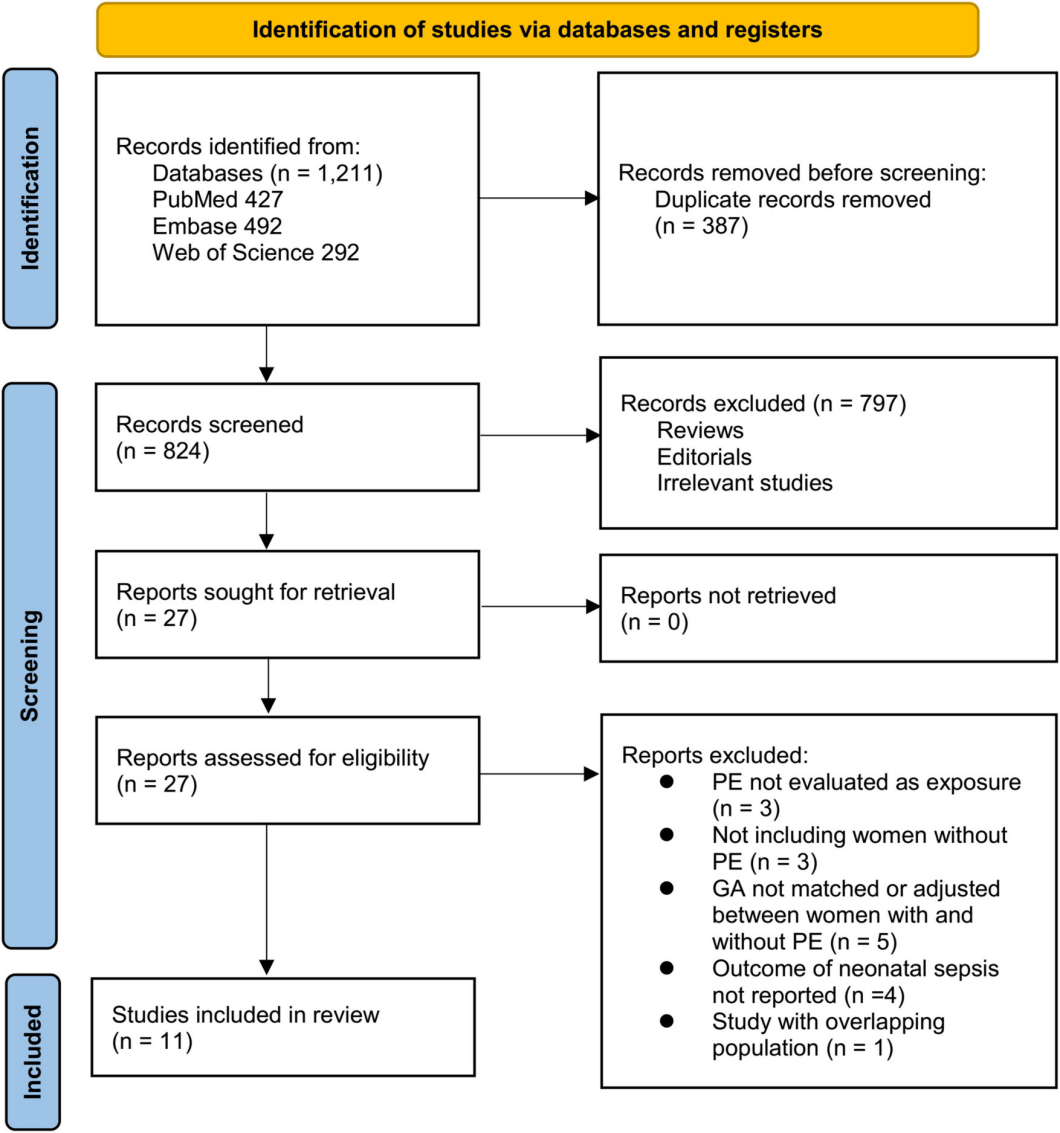
PRISMA flow diagram of the study selection process.

The study selection procedure is illustrated in Figure 1. A total of 1,211 records were retrieved from the three databases, and 387 duplicates were removed. Following screening of titles and abstracts, 797 records were excluded for failing to meet the predefined inclusion criteria. Twenty-seven articles underwent full-text evaluation by two independent reviewers, after which 16 studies were excluded for the reasons detailed in Figure 1. Ultimately, eleven studies met the eligibility criteria and were included in the quantitative meta-analysis (15–25).

### Summary of study characteristics

Table 1 summarizes the characteristics of the included cohort studies. A total of 11 cohort studies were included, comprising five retrospective studies (15–17, 21, 23) and six prospective studies (18–20, 22, 24, 25). These studies were published between 1995 and 2024 and were conducted across North America (USA), Europe (Germany, Denmark, Sweden), Asia (China), and South America (Brazil), reflecting broad geographic representation. The sample sizes varied markedly, ranging from 27 to 805,591 neonates, with a combined population of 1,513,008 participants. The number of neonates born to women with preeclampsia ranged from 18 to 34,145, with a total number of 65,848. Mean GA at delivery varied substantially, from 28.2 to 40.0 weeks, reflecting inclusion of both preterm and term populations. Mean birth weight of neonates born to women with preeclampsia ranged from 0.98 to 3.29 kg. Preeclampsia was generally defined according to standard clinical criteria, including new-onset hypertension (blood pressure ≥140/90 mmHg after 20 weeks of gestation) combined with proteinuria (≥300 mg/24 h), although two recent large population-based studies (24, 25) identified preeclampsia using International Classification of Diseases, 10th Revision (ICD-10) codes. Neonatal sepsis was diagnosed using culture-proven bacteremia in most early and hospital-based studies (15–17, 20, 22), whereas some studies relied on clinically diagnosed sepsis (18, 19, 21) or ICD-10 coding in registry-based cohorts (23–25). The number of neonatal sepsis cases ranged from 3 to 10,598 across studies, with an overall number of 24,925 (1.6%). GA at delivery was matched or adjusted among all the included studies when the association between preeclampsia and neonatal sepsis was analyzed. Ten of the included studies (15–17, 19–25) also controlled other confounders, such as maternal age, body mass index, comorbidities (e.g., diabetes, chronic hypertension), smoking, obstetric factors, perinatal interventions, and birth weight of the neonates, to a varying degree.

**Table 1:** Characteristics of the included cohort studies.

Study	Design	Country	No. of neonates included	Mean maternal age (years)	Mean GA at delivery (weeks)	Diagnostic criteria for PE	No. of neonates born to women with PE	Mean BW of neonates (kg) born to women with PE	Male sex of neonates (%)	Diagnostic methods for neonatal sepsis	No. of cases with neonatal sepsis	Variables matched or adjusted
Friedman et al. (15)	R	USA	446	21	32.7	BP ≥140/90 mmHg (twice, 6 h apart), and proteinuria ≥300 mg/24h	223	1.52	42.6	Culture-proved bacteremia along with characteristic clinical signs	40	GA, race, infant gender, and mode of delivery
Paul et al. (16)	R	USA	504	NR	28.3	New-onset hypertension (≥140/90 mmHg after 20 weeks) and proteinuria (≥300 mg in 24 h urine)	88	1.10	NR	One or more positive blood cultures for bacteria or fungus during hospital course, accompanied by clinical signs. Coagulase-negative staph required clinical signs, response to antibiotics, and treatment ≥7 days.	88	GA, central venous catheter, mechanical ventilation, C-section, PROM, chorioamnionitis, SGA, prenatal MgSO4, and prenatal steroids
Cheng et al. (17)	R	Taiwan (China)	89	NR	28.3	DBP ≥90 mmHg on two occasions or one reading ≥110 mmHg, and proteinuria ≥300 mg/24h	28	0.98	47.2	Culture-proved bacteremia with unstable vital signs	30	GA and birth weight
Tastekin et al. (18)	P	Turkey	27	27.6	32.2	BP ≥140/90 mmHg (twice, 6 h apart) with proteinuria (≥0.3 g/24 h) or pathologic edema	18	1.49	NR	Clinically diagnosed sepsis	3	GA
Cetinkaya et al. (19)	P	Turkey	84	NR	31.3	BP ≥140/90 mmHg and proteinuria ≥300 mg/24 h after 20 weeks’ gestation	51	1.35	41.7	Clinically diagnosed sepsis	32	GA and infant gender
Procianoy et al. (20)	P	Brazil	911	NR	30.5	Hypertension (BP ≥140/90) after 20 weeks with proteinuria (>300 mg/24 h) and edema, with no other cause	308	1.08	49.3	Culture-proved sepsis	246	GA, mechanical ventilation, total parenteral nutrition, central catheter, SGA
Duramaz et al. (21)	R	Turkey	248	NR	31.2	New-onset hypertension (BP ≥140/90) and proteinuria (≥300 mg/24 h) after 20 weeks’ gestation	140	1.59	59.7	Clinically diagnosed sepsis	38	GA, birth weight, neonatal gender, and antenatal corticosteroid
Bossung et al. (21)	P	Germany	16035	NR	28.2	BP ≥140/90 mmHg and proteinuria ≥300 mg/24 h after 20 weeks’ gestation	2652	1.00	51.7	Culture-proved sepsis	1996	GA, antenatal steroids, mode of delivery, gender, birth weight, multiple birth, and IUGR
Harrison et al. (23)	R	USA	1,80,277	27.8	38.7	New-onset hypertension (BP ≥140/90) after 20 weeks+proteinuria (≥300 mg/24 h or 2 + dipstick), with or without organ damage	8331	2.87	NR	ICD-10 codes evidenced	3974	Maternal age, race/ethnicity, insurance, BMI, nulliparity, preexisting diabetes, chronic hypertension, tobacco use, induction of labor, number of vaginal exams, IUPC, FSE, intrapartum chorioamnionitis, mode of delivery, GBS status, PROM, GA at delivery, receipt of steroids, and antibiotics in labor
Havers-Borgersen et al. (24)	P	Denmark	5,08,796	28	40	ICD-10 codes evidenced	19864	3.29	NR	ICD-10 codes evidenced	10,598	Maternal age, GA, and birth weight
Ulfsdottir et al. (25)	P	Sweden	8,05,591	NR	39.5	ICD-10 codes evidenced	34,145	3.10	NR	ICD-10 codes evidenced	7,880	Year of birth, maternal age, maternal height, maternal BMI, GA at delivery, maternal diabetes, maternal kidney disease, maternal SLE, and smoking

NR, not reported; R, retrospective study; P, prospective study; GA, gestational age; PE, preeclampsia; BP, blood pressure; DBP, diastolic blood pressure; BW, birth weight; SGA, small for gestational age; MgSO₄, magnesium sulfate; PROM, premature rupture of membranes; IUGR, intrauterine growth restriction; ICD-10, International Classification of Diseases, 10th Revision; BMI, body mass index; IUPC, intrauterine pressure catheter; FSE, fetal scalp electrode; GBS, Group B Streptococcus; SLE, systemic lupus erythematosus.

### Study quality evaluation

Methodological quality was assessed using the NOS, and detailed results are presented in Table 2. The NOS scores ranged from 7 to 9, indicating generally good methodological quality. Six studies (15–17, 20–22) achieved the maximum score of 9, reflecting strong cohort representativeness, appropriate exposure ascertainment, confirmation that neonatal sepsis was not present at baseline, adequate control for GA at delivery and additional confounders, reliable outcome assessment, and sufficient follow-up. Four studies scored 7 (18, 19, 24, 25), mainly due to limited control for additional confounding factors or less robust exposure/outcome ascertainment, particularly in studies relying on registry-based coding. One study (23) scored 8 because of limitations in outcome assessment despite adequate control of confounders. Importantly, all studies received full stars for cohort selection domains, and all controlled for GA at delivery, which is a critical confounder in the association between preeclampsia and neonatal sepsis. Overall, the included studies were considered to be of moderate to high quality, supporting the robustness and credibility of the pooled estimates examining the association between maternal preeclampsia and the risk of neonatal sepsis.

**Table 2:** Study quality evaluation via the Newcastle-Ottawa scale.

Study	Representativeness of the exposed cohort	Selection of the non-exposed cohort	Ascertainment of exposure	Outcome not present at baseline	Control for GA at delivery	Control for other confounding factors	Assessment of outcome	Enough long follow-up duration	Adequacy of follow-up of cohorts	Total
Friedman et al. (15)	1	1	1	1	1	1	1	1	1	9
Paul et al. (16)	1	1	1	1	1	1	1	1	1	9
Cheng et al. (17)	1	1	1	1	1	1	1	1	1	9
Tastekin et al. (18)	1	1	1	1	1	0	0	1	1	7
Cetinkaya et al. (19)	1	1	1	1	1	0	0	1	1	7
Procianoy et al. (20)	1	1	1	1	1	1	1	1	1	9
Duramaz et al. (21)	1	1	1	1	1	1	0	1	1	9
Bossung et al. (22)	1	1	1	1	1	1	1	1	1	9
Harrison et al. (23)	1	1	1	1	1	1	0	1	1	8
Havers et al. (24)	1	1	0	1	1	1	0	1	1	7
Ulfsdottir et al. (25)	1	1	0	1	1	1	0	1	1	7

### Meta-analysis results

**Figure 2:**
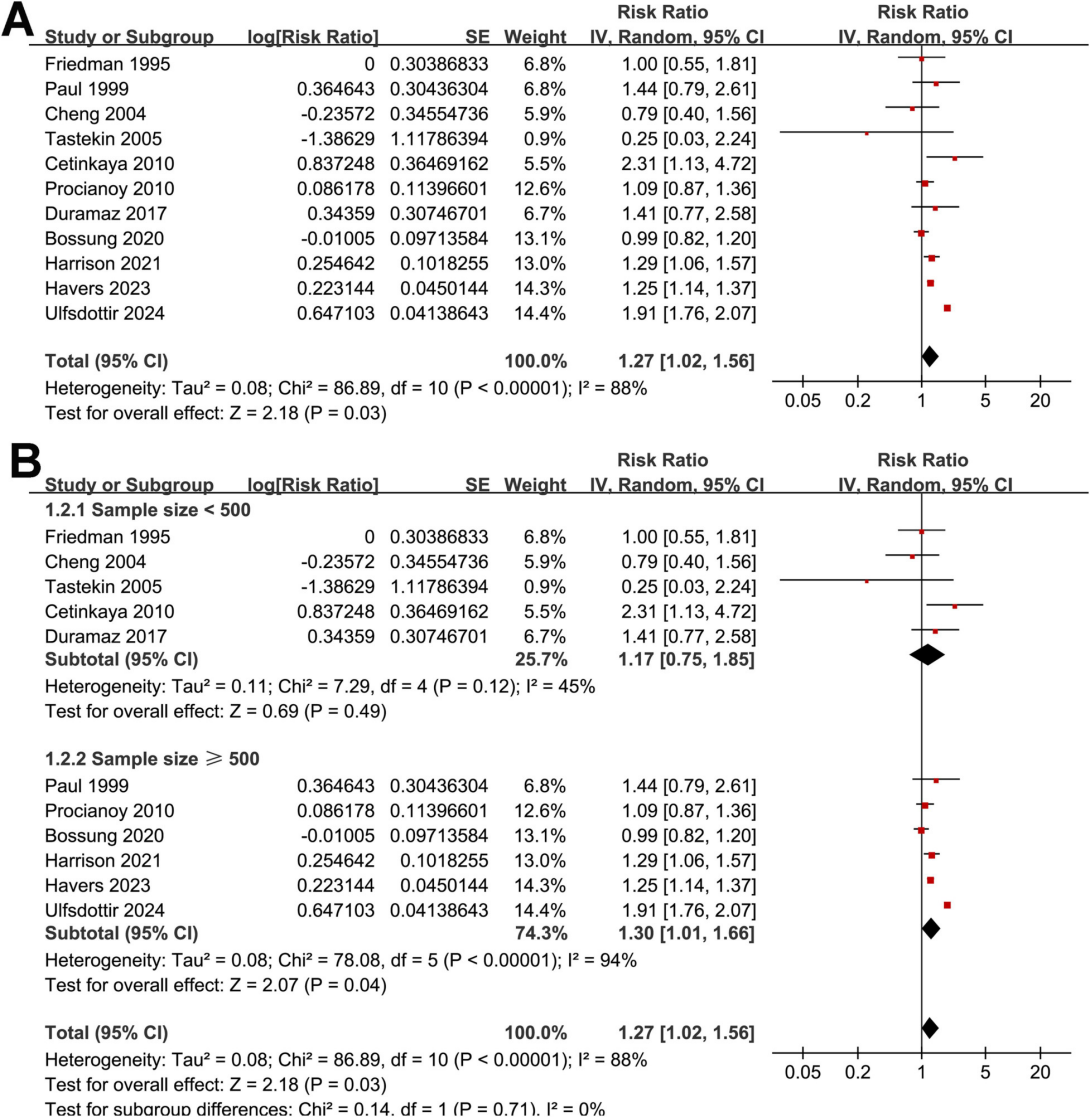
Forest plots showing the meta-analysis of the association between preeclampsia and the risk of neonatal sepsis: **(A)** overall meta-analysis; **(B)** subgroup analysis by sample size.

The pooled analysis of 11 studies (15–25) demonstrated that preeclampsia was significantly associated with an increased risk of neonatal sepsis (RR: 1.27, 95% CI: 1.02–1.56, *p* = 0.03; Figure 2A). However, substantial heterogeneity was observed (Cochrane Q test *p* < 0.001; *I*^2^ = 88%). According to the GRADE assessment, the overall certainty of evidence for the association between maternal preeclampsia and neonatal sepsis was low (Table 3). Leave-one-out sensitivity analyses yielded consistent results, with pooled RRs ranging from 1.18 to 1.32 (all *p* < 0.05), indicating the robustness of the overall estimate. Notably, exclusion of the study with the largest sample size (25) markedly reduced heterogeneity (RR: 1.18, 95% CI: 1.05–1.32, *p* = 0.006; *I*^2^ = 34%), suggesting that this study contributed substantially to between-study variability.

**Table 3:** Summary of findings and certainty of evidence (GRADE).

Outcome	No. of participants (studies)	Study design	Risk of bias	Inconsistency	Indirectness	Imprecision	Publication bias	Relative effect	Certainty of evidence (GRADE)	Comments
Neonatal sepsis	1,513,008 (11 cohort studies)	Observational (cohort)	Not serious—Most studies had moderate to high quality (NOS 7–9) with adjustment for gestational age, although residual confounding cannot be excluded.	Not serious—Although substantial heterogeneity was observed (*I*^2^ = 88%), the direction of effect was consistent across studies, sensitivity analyses were robust, and meta-regression partially explained the variability	Not serious—Population, exposure, comparator, and outcome directly addressed the review question.	Not serious—Confidence interval relatively narrow and statistically significant; large sample size.	Not serious—Funnel plot approximately symmetrical and Egger’s test not significant (*p* = 0.47), although limited power.	RR (95% CI): 1.27 (1.02–1.56)	⊕⊕⦻⦻ Low	Maternal preeclampsia may be associated with a modest increase in neonatal sepsis risk; however, heterogeneity and observational design warrant cautious interpretation.

GRADE, Grading of Recommendations Assessment, Development and Evaluation; RR, risk ratio; CI, confidence interval; NOS, Newcastle–Ottawa Scale.

Specific reasons for each GRADE domain, including: Risk of bias: Downgraded if a significant proportion of studies had unclear or high risk of bias in key domains. Inconsistency: Downgraded if substantial heterogeneity was observed (*I*^2^ > 75%) and could not be explained by subgroup analyses or meta-regression. Indirectness: Evaluated but not downgraded, as all included studies directly assessed the population and outcomes of interest. Imprecision: Downgraded if confidence intervals were wide, or if the overall sample size was small. Publication bias: Assessed using funnel plots and Egger’s test; downgraded if significant asymmetry suggested potential bias.

### Subgroup analysis results

**Figure 3:**
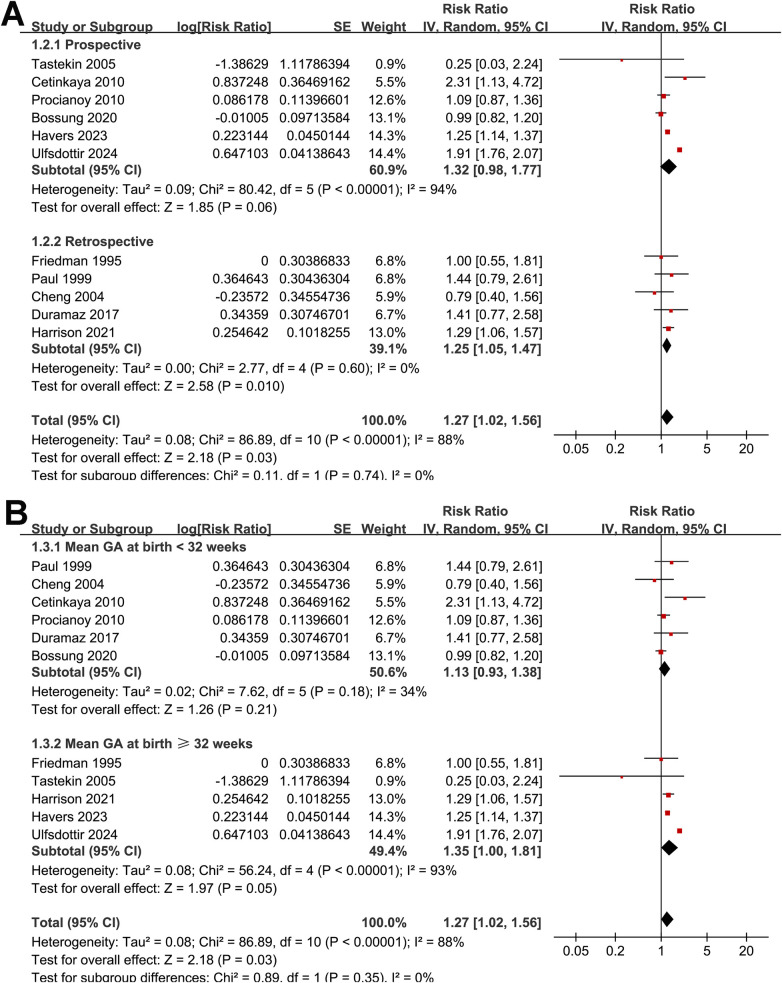
Forest plots for subgroup analyses of the association between preeclampsia and the risk of neonatal sepsis: **(A)** stratified by study design; **(B)** stratified by mean GA at delivery.

**Figure 4:**
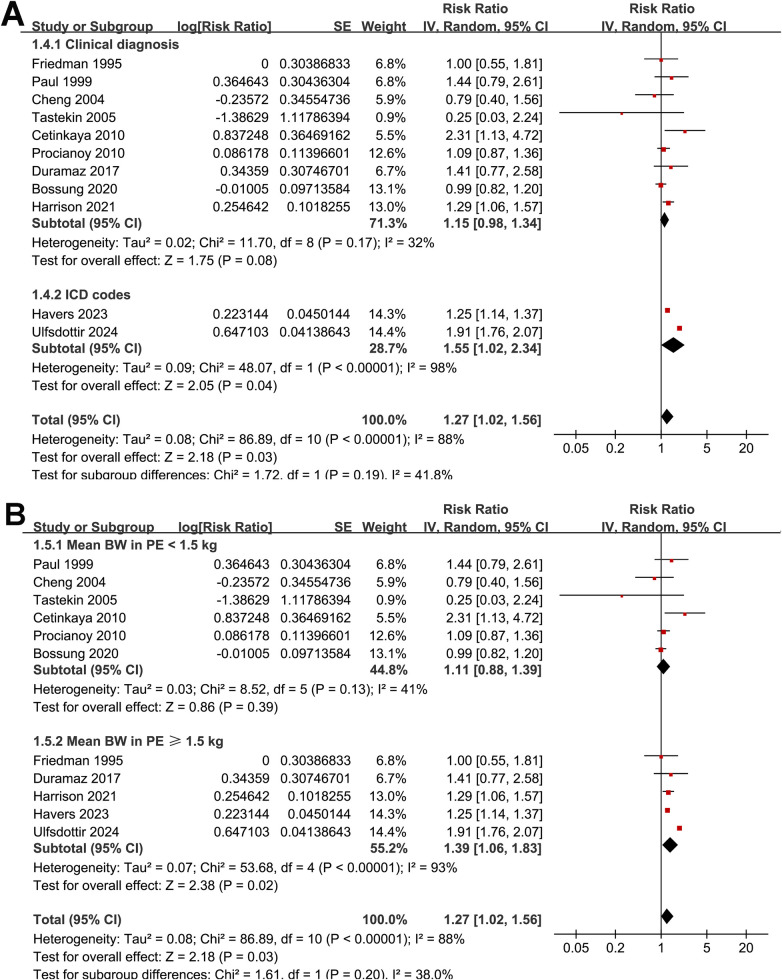
Forest plots for subgroup analyses of the association between preeclampsia and the risk of neonatal sepsis: **(A)** stratified by methods for the diagnosis of preeclampsia; **(B)** stratified by birth weight of neonates born to women with preeclampsia.

**Figure 5:**
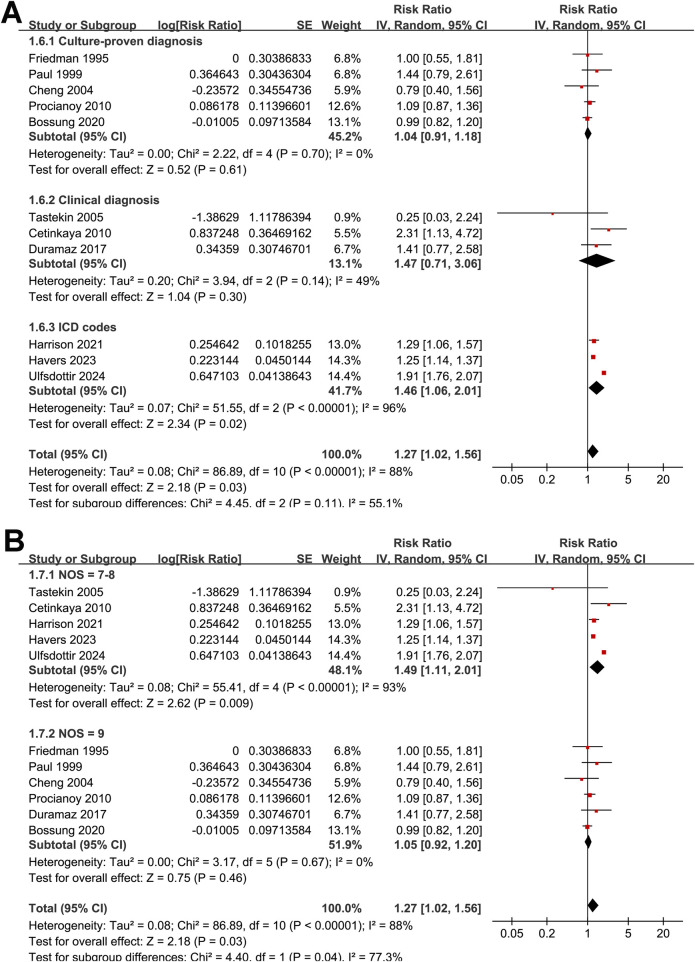
Forest plots for subgroup analyses of the association between preeclampsia and the risk of neonatal sepsis: **(A)** stratified by methods for the diagnosis of neonatal sepsis; **(B)** stratified by NOS scores.

Subgroup analyses did not reveal statistically significant differences in the association according to sample size (<500 vs. ≥500; RR: 1.17 vs. 1.30; *p* for subgroup difference = 0.71; Figure 2B), study design (prospective vs. retrospective; RR: 1.32 vs. 1.25; *p* = 0.74; Figure 3A), mean gestational age at birth (< 32 vs. ≥32 weeks; RR: 1.13 vs. 1.35; *p* = 0.35; Figure 3B), method of preeclampsia ascertainment (clinical diagnosis vs. ICD codes; RR: 1.15 vs. 1.55; *p* = 0.19; Figure 4A), or mean birth weight of neonates born to women with preeclampsia (<1.5 vs. ≥1.5 kg; RR: 1.11 vs. 1.39; *p* = 0.20; Figure 4B). Similarly, no significant subgroup difference was observed according to the diagnostic method for neonatal sepsis (blood culture vs. clinical diagnosis vs. ICD codes; RR: 1.04 vs. 1.47 vs. 1.46; *p* = 0.11; Figure 5A). Interestingly, a stronger association was observed in studies with NOS scores of 7 or 8 compared with those scoring 9 (RR: 1.49 vs. 1.05; *p* for subgroup difference = 0.04; Figure 5B), suggesting that study quality may have influenced the magnitude of the observed association.

### Meta-regression analysis results

The results of the univariate meta-regression analysis are presented in Table 4. Meta-regression showed that sample size was significantly associated with the effect estimate (coefficient: 5.85 × 10^−7^, 95% CI: 1.33 × 10^−7^ to 1.04 × 10^−6^, *p* = 0.02), explaining 71.1% of the between-study heterogeneity (adjusted *R*^2^ = 71.1%). This suggests that study size may have contributed to heterogeneity, with larger registry-based studies exerting greater influence on the pooled estimate. In contrast, mean GA at delivery (coefficient: 0.028, 95% CI: −0.010 to 0.066, *p* = 0.13; adjusted R² = 32.5%) and mean BW of neonates born to women with preeclampsia (coefficient: 0.14, 95% CI: −0.05 to 0.33, *p* = 0.13; adjusted *R*^2^ = 30.6%) were not significantly associated with the effect size. Notably, study quality as assessed by the NOS was inversely associated with the pooled effect estimate (coefficient: −0.19, 95% CI: −0.37 to −0.01, *p* = 0.04), explaining 47.6% of the heterogeneity. This finding indicates that studies with higher methodological quality tended to report weaker associations between preeclampsia and neonatal sepsis.

**Table 4:** Results of univariate meta-regression analysis.

Variables	RR for the association between PE and the risk of neonatal sepsis			
	Coefficient	95% CI	*p* values	Adjusted *R*^2^
Sample size	5.85 × 10^−7^	1.33 × 10^−7^ to 1.04 × 10^−6^	0.02	71.1%
Mean GA at birth	0.028	−0.010 to 0.066	0.13	32.5%
Mean BW of neonates born to women with PE	0.14	−0.05 to 0.33	0.13	30.6%
NOS	−0.19	−0.37 to −0.01	0.04	47.6%

RR, relative risk; PE, preeclampsia; CI, confidence interval; GA, gestational age; BW, birth weight; NOS, Newcastle–Ottawa Scale.

### Publication bias

**Figure 6:**
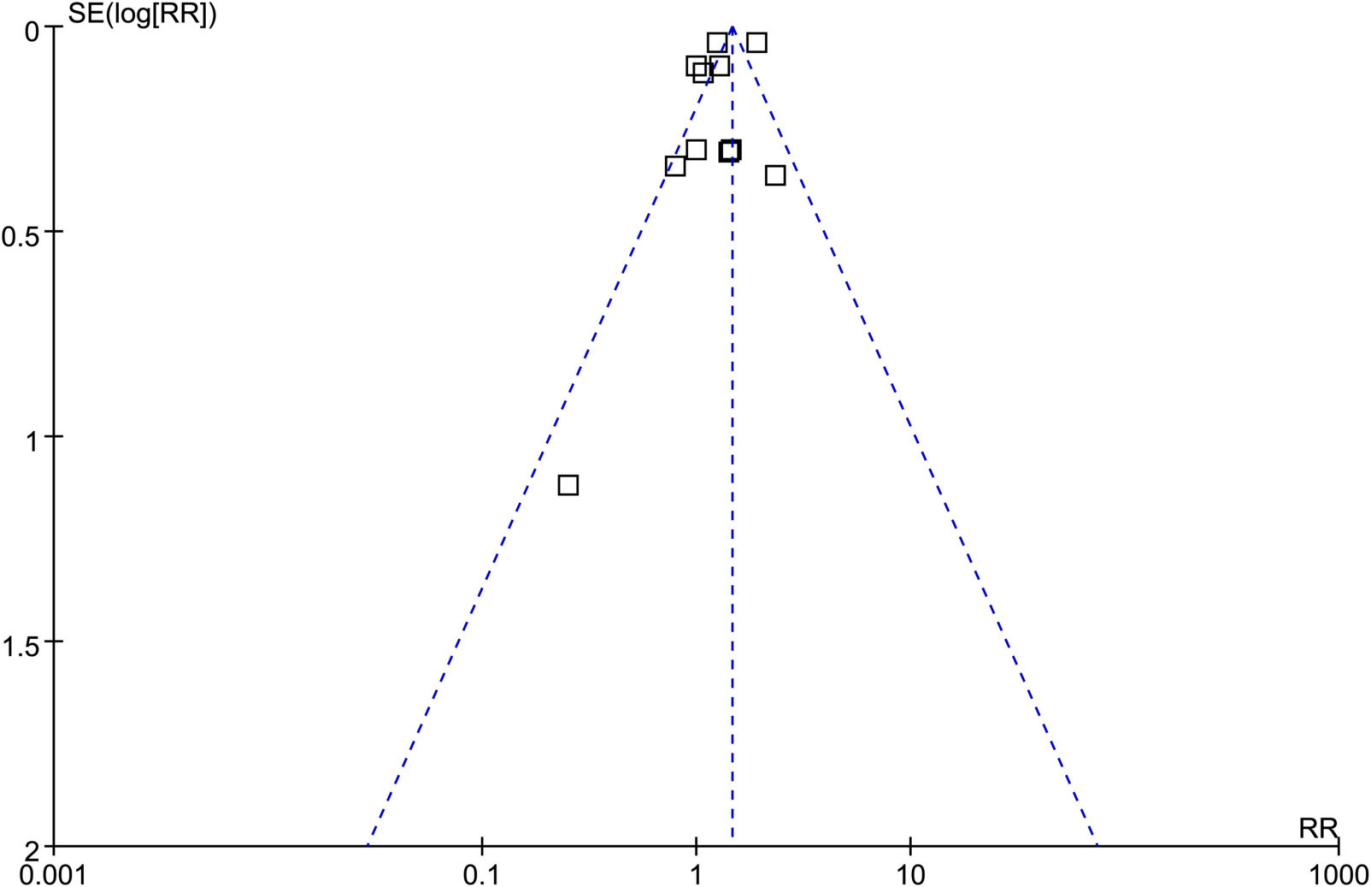
Funnel plots evaluating potential publication bias in the meta-analysis of the association between preeclampsia and the risk of neonatal sepsis.

As shown in Figure 6, the funnel plots for the association between preeclampsia and the risk of neonatal sepsis appeared largely symmetrical. Consistent with this, Egger's test did not indicate statistically significant asymmetry (*p* = 0.47). However, given the relatively small number of included studies (*k* = 11), the power of funnel plot asymmetry tests is limited, and publication bias cannot be definitively excluded; therefore, these findings should be interpreted with caution.

## Discussion

In this meta-analysis of 11 observational cohort studies in which GA at delivery was matched or adjusted, we observed a modest association between maternal preeclampsia and the risk of neonatal sepsis. While the pooled estimate suggested a statistically significant increased risk, substantial heterogeneity was apparent across studies. Sensitivity, subgroup, and meta-regression analyses provided insights into potential sources of variability and underscored the importance of cautious interpretation. Overall, the low certainty of evidence, as assessed by the GRADE framework, reflects the observational nature of the included studies and the presence of between-study heterogeneity.

## Interpretation of findings

The modest association between preeclampsia and neonatal sepsis may be biologically plausible. Preeclampsia is characterized by systemic inflammation, endothelial dysfunction, and altered placental function (33, 34). These pathophysiological changes could influence fetal immune development and compromise neonates' ability to respond effectively to infectious challenges after birth (35). Chronic intrauterine inflammation associated with preeclampsia may prime the fetal immune system toward dysregulation (36), while impaired placental transfer of protective immunoglobulins and immune-modulating factors may leave neonates more vulnerable to sepsis in the early postnatal period (37, 38). Additionally, preeclamptic pregnancies are often complicated by placental insufficiency and oxidative stress, factors that may further impair fetal immune competence (39). Nonetheless, the direct mechanistic link between preeclampsia and neonatal sepsis remains to be fully elucidated in mechanistic and clinical studies.

Interpretation of our results must account for study-level variability. Sensitivity analyses demonstrated that the exclusion of any individual study did not substantially alter the pooled estimate, supporting the robustness of the association. However, exclusion of the largest study (25) markedly reduced heterogeneity, indicating that this large registry-based cohort exerted significant influence on the overall estimate. This observation suggests that study design, population characteristics, and case ascertainment methods may contribute importantly to between-study differences. Indeed, registry-based studies may capture a broader, more heterogeneous population with varying clinical practices and coding accuracy compared with smaller, hospital-based cohorts with prospective data collection.

Subgroup analyses did not reveal statistically significant differences in the association across strata defined by sample size, study design (prospective vs. retrospective), mean GA at delivery (<32 vs. ≥32 weeks), diagnostic methods for preeclampsia (clinical vs. ICD codes), birth weight categories, or diagnostic criteria for neonatal sepsis. The absence of significant subgroup effects suggests that the modest association between preeclampsia and neonatal sepsis may be reasonably consistent across diverse settings and study characteristics. Nevertheless, the lack of statistically significant subgroup differences does not rule out clinically meaningful variation, particularly given the wide confidence intervals and limited number of studies in some subgroups.

Notably, studies with lower methodological quality (NOS scores 7–8) tended to report stronger associations compared with higher-quality studies (NOS score 9). This pattern could reflect residual confounding, differential adjustment for important covariates, or biases inherent in lower-quality designs. Meta-regression supported the influence of study quality on effect estimates, with lower NOS scores associated with larger pooled RRs. Sample size also emerged as a contributor to heterogeneity in meta-regression, suggesting that statistical power and precision may impact effect estimates. These findings highlight the necessity of considering study quality and design features when interpreting pooled results from observational studies.

## Strengths

We conducted a comprehensive and up-to-date literature search across major biomedical databases, reducing the risk of missing relevant studies. We included only cohort studies in which GA at delivery—one of the most potent confounders for neonatal sepsis—was either matched or adjusted, enhancing the internal validity of the pooled estimate. We employed multiple analytic strategies, including sensitivity, subgroup, and meta-regression analyses, to explore heterogeneity and assess the robustness of findings. These additional analyses enabled a more nuanced interpretation than reliance on pooled estimates alone. Furthermore, we evaluated study quality formally using the NOS and incorporated quality assessment into our interpretation of results.

## Limitations

Despite these strengths, our meta-analysis has important limitations. First, several included studies were retrospective in design (15–17, 21, 23), raising the possibility of recall bias, selection bias, and incomplete adjustment for confounders. Although we restricted inclusion to studies controlling for GA at delivery, residual confounding by unmeasured factors such as socioeconomic status, maternal comorbidities, antenatal steroid use, mode of delivery, or intrapartum antibiotic exposure cannot be excluded (40). Second, substantial heterogeneity was observed across studies, likely reflecting differences in population characteristics, clinical practices, and outcome definitions. Because individual participant data (IPD) were not available, we could not standardize definitions of exposures, outcomes, or covariates across studies or perform more granular stratified analyses. Third, some studies relied on ICD codes to identify both preeclampsia and neonatal sepsis (24, 25). While administrative data facilitate large sample sizes, diagnostic coding may be subject to misclassification and variable validity, which could bias effect estimates toward or away from the null. Fourth, although we explored study quality using NOS stratification, the NOS itself has limitations and may not capture all aspects of methodological rigor relevant to neonatal infection research (41). In addition, although we assessed publication bias using funnel plots and Egger's test, the relatively small number of included studies (*k* = 11) limits the reliability and statistical power of these methods. Therefore, the possibility of publication bias cannot be fully excluded. Moreover, only studies published in English were included, which may introduce language bias and result in the exclusion of potentially relevant studies published in other languages. Besides, although we searched three major databases (PubMed, Embase, and Web of Science), additional sources such as grey literature and regional databases were not systematically searched, which may have resulted in the omission of some relevant studies. Finally, observational studies can identify associations but cannot establish causality. The modest association observed here should be interpreted as hypothesis-generating rather than definitive evidence of a causal link between preeclampsia and neonatal sepsis.

## Clinical implications/future directions

The clinical implications of our findings should be considered in the context of these limitations. If preeclampsia does indeed confer even a modest increase in sepsis risk, clinicians managing neonates born to preeclamptic mothers may consider heightened surveillance for signs of infection, particularly in the early postnatal period. However, standardized clinical guidelines for sepsis screening in this specific subgroup are lacking, and routine interventions based solely on maternal preeclampsia cannot be advocated without stronger evidence. Future research should aim to elucidate the biological mechanisms linking preeclampsia to neonatal immune vulnerability, ideally through prospective cohort studies with detailed phenotyping, standardized infection definitions, and comprehensive adjustment for confounders. Integration of individual-level data across studies or establishment of large multi-center prospective registries could enable more precise estimation and investigation of effect modification by factors such as GA, birth weight, antenatal interventions, and neonatal care practices.

## Conclusions

In conclusion, this systematic review and meta-analysis suggests that maternal preeclampsia may be associated with a modestly increased risk of neonatal sepsis after accounting for gestational age at delivery. This finding may have clinical implications, as neonates born to mothers with preeclampsia could benefit from heightened surveillance for early signs of infection, although causality cannot be established. By focusing on studies with gestational age–matched or adjusted analyses, this study provides a more robust evaluation of this association and helps to clarify previously inconsistent findings in the literature. However, substantial heterogeneity and the observational nature of the included studies limit the certainty of the evidence. Future research should prioritize well-designed prospective studies with standardized definitions of neonatal sepsis, detailed adjustment for confounders, and, where possible, individual participant data analyses to better elucidate the underlying mechanisms and clinical significance of this association.

## Data Availability

The original contributions presented in the study are included in the article/Supplementary Material, further inquiries can be directed to the corresponding author.

## References

[1] Strunk T Molloy EJ Mishra A Bhutta ZA. Neonatal bacterial sepsis. Lancet. (2024) 404(10449):277–93. 10.1016/S0140-6736(24)00495-1 38944044

[2] Flannery DD Ramachandran V Schrag SJ. Neonatal early-onset sepsis: epidemiology, microbiology, and controversies in practice. Clin Perinatol. (2025) 52(1):15–31. 10.1016/j.clp.2024.10.002 39892950

[3] Dramowski A Bolton L Fitzgerald F Bekker A. Neonatal sepsis in low- and middle-income countries: where are we now? Pediatr Infect Dis J. (2025) 44(6):e207–10. 10.1097/INF.0000000000004815 40168607 PMC7617557

[4] Flannery DD Puopolo KM. Neonatal early-onset sepsis. Neoreviews. (2022) 23(11):756–70. 10.1542/neo.23-10-e756 36316253

[5] Coggins SA Glaser K. Updates in late-onset sepsis: risk assessment, therapy, and outcomes. Neoreviews. (2022) 23(11):738–55. 10.1542/neo.23-10-e738 36316254 PMC9675597

[6] Zasada M Kwinta P Durlak W Bik-Multanowski M Madetko-Talowska A Pietrzyk JJ. Development and maturation of the immune system in preterm neonates: results from a whole genome expression study. Biomed Res Int. (2014) 2014:498318. 10.1155/2014/498318 24982884 PMC4058491

[7] Guo L Han W Su Y Wang N Chen X Ma J Perinatal risk factors for neonatal early-onset sepsis: a meta-analysis of observational studies. J Matern Fetal Neonatal Med. (2023) 36(2):2259049. 10.1080/14767058.2023.2259049 37743349

[8] Kariniotaki C Thomou C Gkentzi D Panteris E Dimitriou G Hatzidaki E. Neonatal sepsis: a comprehensive review. Antibiotics (Basel). (2025) 14(1):6. 10.3390/antibiotics14010006 PMC11761862 39858292

[9] Chappell LC Cluver CA Kingdom J Tong S. Pre-eclampsia. Lancet. (2021) 398(10297):341–54. 34051884 10.1016/S0140-6736(20)32335-7

[10] Roberts JM. Preeclampsia epidemiology(ies) and pathophysiology(ies). Best Pract Res Clin Obstet Gynaecol. (2024) 94:102480. 10.1016/j.bpobgyn.2024.102480 38490067

[11] Martini C Saeed Z Simeone P Palma S Ricci M Arata A Preeclampsia: insights into pathophysiological mechanisms and preventive strategies. Am J Prev Cardiol. (2025) 23:101054. 10.1016/j.ajpc.2025.101054 40703703 PMC12284657

[12] Bokslag A van Weissenbruch M Mol BW de Groot CJ. Preeclampsia; short and long-term consequences for mother and neonate. Early Hum Dev. (2016) 102:47–50. 10.1016/j.earlhumdev.2016.09.007 27659865

[13] Deer E Herrock O Campbell N Cornelius D Fitzgerald S Amaral LM The role of immune cells and mediators in preeclampsia. Nat Rev Nephrol. (2023) 19(4):257–70. 10.1038/s41581-022-00670-0 36635411 PMC10038936

[14] Aouache R Biquard L Vaiman D Miralles F. Oxidative stress in preeclampsia and placental diseases. Int J Mol Sci. (2018) 19(5):1496. 10.3390/ijms19051496 29772777 PMC5983711

[15] Friedman SA Schiff E Kao L Sibai BM. Neonatal outcome after preterm delivery for preeclampsia. Am J Obstet Gynecol. (1995) 172(6):1785–8; discussion 8–92. 10.1016/0002-9378(95)91412-9 7778633

[16] Paul DA Leef KH Sciscione A Tuttle DJ Stefano JL. Preeclampsia does not increase the risk for culture proven sepsis in very low birth weight infants. Am J Perinatol. (1999) 16(7):365–72. 10.1055/s-2007-993886 10614705

[17] Cheng SW Chou HC Tsou KI Fang LJ Tsao PN. Delivery before 32 weeks of gestation for maternal pre-eclampsia: neonatal outcome and 2-year developmental outcome. Early Hum Dev. (2004) 76(1):39–46. 10.1016/j.earlhumdev.2003.10.004 14729161

[18] Tastekin A Ors R Demircan B Saricam Z Ingec M Akcay F. Oxidative stress in infants born to preeclamptic mothers. Pediatr Int. (2005) 47(6):658–62. 10.1111/j.1442-200x.2005.02146.x 16354220

[19] Çetinkaya M Özkan H Köksal N Karali Z Özgür T. Neonatal outcomes of premature infants born to preeclamptic mothers. J Matern Fetal Neonatal Med. (2010) 23(5):425–30. 10.3109/14767050903184173 19670043

[20] Procianoy RS Silveira RC Mussi-Pinhata MM Souza Rugolo LM Leone CR de Andrade Lopes JM Sepsis and neutropenia in very low birth weight infants delivered of mothers with preeclampsia. J Pediatr. (2010) 157(3):434–8. 10.1016/j.jpeds.2010.02.066 20400101

[21] Duramaz B Bilgin L Salihoglu Ö Ertaş K Hatipoğlu S. Neonatal outcomes of preterm infants born to preeclamptic mothers. Marmara Med J. (2017) 30(1):8–13. 10.5472/marumj.299376

[22] Bossung V Fortmann MI Fusch C Rausch T Herting E Swoboda I Neonatal outcome after preeclampsia and HELLP syndrome: a population-based cohort study in Germany. Front Pediatr. (2020) 8:579293. 10.3389/fped.2020.579293 33154958 PMC7586782

[23] Harrison RK Palatnik A. The association between preeclampsia and ICD diagnosis of neonatal sepsis. J Perinatol. (2021) 41(3):460–7. 10.1038/s41372-020-00774-0 32788618

[24] Havers-Borgersen E Fosbøl E Johansen M Køber L Morris JM Seeho SKM. Pre-eclampsia in a first pregnancy and subsequent pregnancy outcomes: a nationwide cohort study. J Epidemiol Community Health. (2023) 77(11):694–703. 10.1136/jech-2023-220829 37541773

[25] Ulfsdottir H Grandahl M Björk J Karlemark S Ekéus C. The association between pre-eclampsia and neonatal complications in relation to gestational age. Acta Paediatr. (2024) 113(3):426–33. 10.1111/apa.17080 38140818

[26] Dimitriadis E Rolnik DL Zhou W Estrada-Gutierrez G Koga K Francisco RPV Pre-eclampsia. Nat Rev Dis Primers. (2023) 9(1):8. 10.1038/s41572-023-00417-6 36797292

[27] Page MJ Moher D Bossuyt PM Boutron I Hoffmann TC Mulrow CD PRISMA 2020 Explanation and elaboration: updated guidance and exemplars for reporting systematic reviews. Br Med J. (2021) 372:n160. 10.1136/bmj.n160 33781993 PMC8005925

[28] Higgins J Thomas J Chandler J Cumpston M Li T Page M Cochrane Handbook for Systematic Reviews of Interventions Version 6.2. London: The Cochrane Collaboration (2021). Available online at: www.training.cochrane.org/handbook

[29] Wells GA Shea B O'Connell D Peterson J Welch V Losos M The Newcastle-Ottawa Scale (NOS) for assessing the quality of nonrandomised studies in meta-analyses (2010). Available online at: http://www.ohri.ca/programs/clinical_epidemiology/oxford.asp (Accessed January 31, 2026).

[30] Higgins JP Thompson SG. Quantifying heterogeneity in a meta-analysis. Stat Med. (2002) 21(11):1539–58. 10.1002/sim.1186 12111919

[31] Marušić MF Fidahić M Cepeha CM Farcaș LG Tseke A Puljak L. Methodological tools and sensitivity analysis for assessing quality or risk of bias used in systematic reviews published in the high-impact anesthesiology journals. BMC Med Res Methodol. (2020) 20(1):121. 10.1186/s12874-020-00966-4 32423382 PMC7236513

[32] Egger M Davey Smith G Schneider M Minder C. Bias in meta-analysis detected by a simple, graphical test. Br Med J. (1997) 315(7109):629–34. 10.1136/bmj.315.7109.629 9310563 PMC2127453

[33] Kutllovci Hasani K Ajeti N Goswami N. Understanding preeclampsia: cardiovascular pathophysiology, histopathological insights and molecular biomarkers. Med Sci (Basel). (2025) 13(3):154. 40981151 10.3390/medsci13030154 PMC12452302

[34] Michalczyk M Celewicz A Celewicz M Woźniakowska-Gondek P Rzepka R. The role of inflammation in the pathogenesis of preeclampsia. Mediators Inflamm. (2020) 2020:3864941. 10.1155/2020/3864941 33082708 PMC7556088

[35] Stojanovska V Scherjon SA Plösch T. Preeclampsia as modulator of offspring health. Biol Reprod. (2016) 94(3):53. 10.1095/biolreprod.115.135780 26792940

[36] Cornelius DC. Preeclampsia: from inflammation to immunoregulation. Clin Med Insights Blood Disord. (2018) 11:1179545X17752325. 10.1177/1179545X17752325 29371787 PMC5772493

[37] Beudeker CR Vijlbrief DC van Montfrans JM Rooijakkers SHM van der Flier M. Neonatal sepsis and transient immunodeficiency: potential for novel immunoglobulin therapies? Front Immunol. (2022) 13:1016877. 10.3389/fimmu.2022.1016877 36330515 PMC9623314

[38] Wynn JL Wong HR. Pathophysiology of neonatal sepsis. Fetal Neonatal Physiol. (2017):1536–52.e10. 10.1016/B978-0-323-35214-7.00152-9

[39] Sultana Z Maiti K Aitken J Morris J Dedman L Smith R. Oxidative stress, placental ageing-related pathologies and adverse pregnancy outcomes. Am J Reprod Immunol. (2017) 77(5):e12653. 10.1111/aji.12653 28240397

[40] Vulcănescu A Siminel MA Dinescu SN Boldeanu MV Dijmărescu AL Manolea MM Systematic review: maternal risk factors, socioeconomic influences, neonatal biomarkers and management of early-onset sepsis in late preterm and term newborns-A focus on European and eastern European contexts. Life (Basel). (2025) 15(2):292. 40003700 10.3390/life15020292 PMC11856718

[41] Johnson JL Adkins D Chauvin S. A review of the quality indicators of rigor in qualitative research. Am J Pharm Educ. (2020) 84(1):7120. 10.5688/ajpe7120 32292186 PMC7055404

