# Supplementary material for: Association between maternal preeclampsia and the risk of neonatal sepsis: a systematic review and meta-analysis
Source: Front Pediatr. 2026 Apr 13;14:1813564. doi: 10.3389/fped.2026.1813564 (PMC13111289; doi:10.3389/fped.2026.1813564)
Supplement: Supplementary file 2 [file Table2.docx]

**Detailed search strategy for each database**

**PubMed**

("Pre-Eclampsia"[Mesh] OR preeclampsia[tiab] OR "pre-eclampsia"[tiab] OR preeclamptic[tiab]) AND ("Infant, Newborn"[Mesh] OR "Infant"[Mesh] OR neonatal[tiab] OR newborn*[tiab] OR infant*[tiab] OR neonat*[tiab]) AND ("Sepsis"[Mesh] OR "Septicemia"[Mesh] OR sepsis[tiab] OR septic[tiab] OR septicemia[tiab])

**Embase**

('preeclampsia'/exp OR preeclampsia:ti,ab OR 'pre-eclampsia':ti,ab OR preeclamptic:ti,ab) AND ('newborn'/exp OR 'infant'/exp OR neonatal:ti,ab OR newborn*:ti,ab OR infant*:ti,ab OR neonat*:ti,ab) AND ('sepsis'/exp OR 'septicemia'/exp OR sepsis:ti,ab OR septic:ti,ab OR septicemia:ti,ab)

**Web of Science**

TS = ((preeclampsia OR "pre-eclampsia" OR preeclamptic) AND (neonatal OR newborn* OR infant* OR neonat*) AND (sepsis OR septic OR septicemia))
